# Identification of a Novel Pattern Recognition Receptor DM9 Domain Containing Protein 4 as a Marker for Pro-Hemocyte of Pacific Oyster *Crassostrea gigas*

**DOI:** 10.3389/fimmu.2020.603270

**Published:** 2021-02-12

**Authors:** Zhihao Jia, Shuai Jiang, Mengqiang Wang, Xiudan Wang, Yu Liu, Zhao Lv, Xiaorui Song, Yiqun Li, Lingling Wang, Linsheng Song

**Affiliations:** ^1^ Liaoning Key Laboratory of Marine Animal Immunology, Dalian Ocean University, Dalian, China; ^2^ Key Laboratory of Experimental Marine Biology, Institute of Oceanology, Chinese Academy of Sciences, Qingdao, China; ^3^ Liaoning Key Laboratory of Marine Animal Immunology and Disease Control, Dalian Ocean University, Dalian, China; ^4^ Functional Laboratory of Marine Fisheries Science and Food Production Process, Qingdao National Laboratory for Marine Science and Technology, Qingdao, China; ^5^ Southern Laboratory of Ocean Science and Engineering (Guangdong, Zhuhai), Zhuhai, China

**Keywords:** *Crassostrea gigas*, DM9 domain, pattern recognition, innate immune response, pro-hemocytes

## Abstract

DM9 refers to an uncharacterized protein domain that is originally discovered in *Drosophila melanogaster*. Two proteins with DM9 repeats have been recently identified from Pacific oyster *Crassostrea gigas* as mannose-specific binding pattern-recognition receptors (PRRs). In the present study, a novel member of DM9 domain containing protein (designated as *Cg*DM9CP-4) was identified from *C. gigas*. *Cg*DM9CP-4, about 16 kDa with only two tandem DM9 domains, was highly enriched in hemocytes and gill. The transcripts level of *Cg*DM9CP-4 in circulating hemocytes were decreased after LPS, PGN and *Vibrio splendidus* stimulations. The recombinant protein of *Cg*DM9CP-4 (r*Cg*DM9CP-4) displayed a broad binding spectrum towards various pathogen-associated molecular patterns (PAMPs) (LPS, PGN, β-glucan and Mannose) and microorganisms (*Staphylococcus aureus*, *Micrococcus luteus*, *V. splendidus*, *V. anguillarum*, *Escherichia coli*, *Pichia pastoris* and *Yarrowia lipolytica*). *Cg*DM9CP-4 was mostly expressed in gill and some of the hemocytes. Flow cytometry analysis demonstrated that the *Cg*DM9CP-4-positive hemocytes accounted for 7.3% of the total hemocytes, and they were small in size and less in granularity. *Cg*DM9CP-4 was highly expressed in non-phagocytes (~82% of total hemocytes). The reactive oxygen species (ROS) and the expression levels of cytokines in *Cg*DM9CP-4-positive hemocytes were much lower than that in *Cg*DM9CP-4-negative hemocytes. The mRNA expression level of *Cg*DM9CP-4 in hemocytes was decreased after RNAi of hematopoietic-related factors (*Cg*GATA, *Cg*Runt, *Cg*SCL, and *Cg*Notch). In addition, *Cg*DM9CP-4-positive cells were found to be much more abundant in hemocytes from gill than that from hemolymph, with most of them located in the gill filament. All these results suggested that *Cg*DM9CP-4 was a novel member of PRR that expressed in undifferentiated pro-hemocytes to mediate immune recognition of pathogens.

## Introduction

Innate immunity is the first line for organism to defense against infections (1, 2). Pattern recognition between the PRRs and the PAMPs is the most crucial step for innate immune response (3). PRRs, such as Lectins, peptidoglycan recognition proteins (PGRPs), gram-negative binding proteins (GNBPs), galectins, thioester-containing proteins (TEPs), scavenger receptors (SRs) and Toll-like receptors (TLRs) are germline-encoded receptors that recognize the conserved PAMPs such as lipopolysaccharides (LPS), peptidoglycan (PGN), glucans, and nucleic acid (4, 5). In invertebrate, an increasing number of PRRs have been characterized with different binding specificity and functions (6). They can serve as opsonins (7, 8), receptors (9, 10) and initiators (11) to facilitate phagocytosis, transduce immune signals and induce clotting and melanization, or involve in other protein modification cascades implicated in different immune reactions (12).

PRRs on the surface of cells are linked with the specific functions of different types of cells and they are widely used in cell typing (13). In mammals, different immunophenotypings of blood cells can be distinguished in terms of cell surface receptors (14). For instance, CD14, which is a specific receptor for LPS, has been employed as a useful marker molecule for monocytes and macrophages (15, 16). There are a variety of lectins expressed on the surface of macrophages, which are corresponding to the differentiation states of the cells (17, 18). In *Drosophila melanogaster*, different marker proteins have been identified from subpopulations of hemocytes (19, 20). Signature proteins for hematopoietic tissue (HPT) cells, semigranular cells (SGC) and granular cells (GC) have also been characterized from crayfish (21). Even though the hematopoietic stem cells have been identified in oyster gill and the hemocytes have been characterized and classified into Agranulocytes, SGC and GC according to the morphological features (22–24), the cell surface markers for hemocytes classification are still very limited in *C. gigas*.

The DM9 domain is firstly identified from *D. melanogaster* with unknown function (25). Proteins with DM9 domain are mainly found in Arthropods and Platyhelminthes and only occasionally in other eukaryotes or prokaryotes (26). Increasing number of DM9 domain containing proteins (DM9CPs) have been discovered in both vertebrates and invertebrates serving as important participators in the immune response (27, 28). DM9CPs are previously named as Natterin because DM9 domain was characterized from the venom of *Thalassophryne nattereri* fish as a new class of proteins with kininogenase activity (29). However, the kininogenase activity of Natterin proteins is mediated by the C-terminal pore-forming toxin-like domain rather than DM9 domain (30). In *C. gigas*, the first DM9-only protein with two tandem DM9 domains is found to be abundant in hemolymph and served as a powerfully mannose binding PRR (31, 32). *Cg*DM9CP-2 with two tandem DM9 domains also participates in the antimicrobial activity as a mannose binding PRR (33). All these evidences emphasize the important role of DM9CPs in mollusk immunity.

The Pacific oyster *C. gigas* is one of dominant aquaculture bivalves worldwide. However, the frequent outbreaks of disease caused by bacterial pathogen cause significant loss of regular production and the economic viability of the industry. Thus, it is of vital importance to study the innate immune system of the oyster. In the present study, a new DM9 domain containing protein (*Cg*DM9CP-4) with two tandem DM9 domains was identified from *C. gigas* with high sequence similarity with *Cg*DM9CP-1 and *Cg*DM9CP-2 (34). Objectives of the present study were to (1) detect the distribution of *Cg*DM9CP-4 in tissues and hemocytes, as well as the temporal expression profiles in hemocytes post various immune challenges, (2) determine its pathogen recognition activity and specific function in hemocytes, and (3) further cognize the involvement of DM9CPs in maturation and differentiation of mollusk hemocytes.

## Materials and Methods

### Oyster, Bacterial Challenge and Tissue Collection

Adult pacific oysters *C. gigas*, about two years old (average shell length of 13.0 cm), were collected from a commercial farm in Qingdao, Shandong Province, China, and cultured in filtered aerated seawater at 18°C for a week before processing.

Different tissues, including gonad, muscle, mantle, gill, and hepatopancreas, were obtained from six adult oysters as parallel samples. Hemolymph from the oysters was collected and immediately centrifuged at 800 × *g*, 4°C for 10 min to harvest the hemocytes. All these samples were stored at -80°C using Trizol reagent (Invitrogen) for subsequent RNA extraction.

After the acclimatization, two hundred oysters were randomly divided into five groups with forty individuals in each group and kept in aerated tanks. Each oyster in the four treated groups received an injection of 100 μL live *V. splendidus* in sterile sea water suspension (1×10^9^ CFU mL^-1^), 100 μL LPS from *E. coli* 0111:B4 (Sigma-Aldrich, 0.5 mg mL^-1^ in sterile sea water), 100 μL of PGN from *S. aureus* (Sigma-Aldrich, 0.8 mg mL^-1^ in sterile sea water), sterile sea water, respectively. The untreated oysters were employed as blank group. After treatment, the oysters were returned to water tanks and six individuals from each group were randomly sampled at 3, 6, 9, 12, 24, and 96 h post-injection, respectively. The hemolymph was collected from the oyster and centrifuged at 800 × *g*, 4°C for 10 min to harvest the hemocytes. All these samples were stored at -80°C using Trizol reagent for subsequent RNA extraction. The gills were also collected from the *V. splendidus* and sterile sea water stimulated oysters at 0, 3, 6, 9, 12, and 24 h for subsequent RNA extraction.

### RNA Isolation and cDNA Synthesis

Total RNA was extracted from samples using Trizol reagent. First-strand cDNA synthesis was carried out based on Promega M-MLV RT Usage information using the DNase I (Promega)-treated total RNA as template and oligo (dT)-adaptor (**Supplementary Table 1**). The synthesis reaction was performed at 42°C for 1 h, terminated by heating at 95°C for 5 min. The cDNA mix was diluted to 1:50 and stored at −80°C for subsequent gene cloning and SYBR Green fluorescent quantitative real-time PCR (qRT-PCR).

### Cloning and Sequence Analysis of Full-Length cDNA

Sequence information of *Cg*DM9CP-4 (JH818057, EKC17431.1) was retrieved from the National Center for Biotechnology Information (http://www.ncbi.nlm.nih.gov/). A pair of gene specific primers, P1 and P2 (**Supplementary Table 1**), were designed to amplify the full-length cDNA sequence of *Cg*DM9CP-4. The PCR product was gel-purified, cloned into the pMD 19-T simple vector (TaKaRa), and sequenced by sequencing primers (**Supplementary Table 1**).

The EditSeq module of DNAStar Laser gene software suite 11.0.0 was used to analyze the deduced amino acid sequence of *Cg*DM9CP-4. The protein motif feature of *Cg*DM9CP-4 was predicted by Simple Modular Architecture Research Tool (SMART) 7.0 (http://smart.embl-heidelberg.de/). ClustalW multiple alignment program 1.81 and multiple alignment show program 2.0 (http://www.bioinformatics.org/sms2/) were used to perform the multiple sequence alignment of DM9 domain containing proteins.

### Real-Time PCR Analysis of *Cg*DM9CP-4 mRNA Expression

*Cg*DM9CP-4 mRNA transcripts were detected by SYBR Green fluorescent qRT-PCR. Two gene specific primers for *Cg*DM9CP-4, P3 and P4 (**Supplementary Table 1**), were used to amplify a fragment of 134 bp. The 162 bp long fragment of oyster Elongation Factor 1 alpha (EF1-a, GeneBank: AB122066), amplified with primers P5 and P6 (**Supplementary Table 1**), was chosen as internal reference. A standard curve was made to detect the efficiencies of the primers. The slope of the regression between the log values and the average Ct values were used to o calculate primer efficiency values as: [10^(−1/The Slope Value) −1]*100. The efficiencies of *Cg*DM9CP-4 and oyster EF were 95% and 98%, respectively.

The SYBR Green qRT-PCR assay was carried out in an ABI PRISM 7500 Sequence Detection System (Applied Biosystems) according to the manual. Dissociation curve analysis of amplification products was performed at the end of each PCR to confirm that only one PCR product was amplified and detected. The relative expression of *Cg*DM9CP-4 was analyzed by the 2^-ΔΔCT^ method. All the data were analyzed by using the SDS 2.0 software (Applied Biosystems) and given in terms of relative mRNA expressed as mean ± SD (N = 4).

### Prokaryotic Expression and Purification of the Recombinant *Cg*DM9CP-4

The completed cDNA fragment of *Cg*DM9CP-4 was amplified with the primers P7 and P8 (**Supplementary Table 1**) with *Nde*I and *Xhol*I (NEB) cleavage site sequences added to the 5’ end, respectively. The PCR fragment was digested by restriction enzymes *Nde*I and *Xhol*I, and ligated into the same restriction enzymes sites of expression vector pET-30a (Novagen). The recombinant plasmid (pET-30a-*Cg*DM9CP-4) was transformed into *E. coli* Transetta (DE3) (TransGen). The positive transformants were incubated in LB medium containing 50 μg mL^-1^ kanamycin at 37°C with shaking at 220 rpm for 4 h. When the culture mediums reached OD_600_ of 0.5–0.7, IPTG was added to the LB medium at a final concentration of 1 mmol L^-1^, and incubated at 16°C with shaking at 180 rpm for 20 h. The bacterial culture was sonicated and centrifuged to get the supernatant containing soluble target protein. The recombinant protein of *Cg*DM9CP-4 (r*Cg*DM9CP-4) was purified by Ni^2+^ chelating Sepharose column (Roche), and the purified protein was dialyzed out of imidazole against ddH_2_O at 4°C for 24 h. The protein was resolved by 12% SDS-polyacrylamide gel electrophoresis (SDS-PAGE), and visualized with Coomassie Bright Blue R250. The concentration of purified soluble protein was quantified by BCA method. The obtained protein was stored at -80°C for subsequent experiment.

### Preparation of Polyclonal Antibody and Western Blotting Analysis

The purified r*Cg*DM9CP-4 was immuned to 6 weeks old rats with a weight of approximately 120 g to acquire polyclonal antibody. Briefly, r*Cg*DM9CP-4 of 1mg mL^-1^ was mixed with freund’s adjuvant to immunize the female rats for four times.

After SDS-PAGE, the samples of r*Cg*DM9CP-4 were electrophoretically transferred onto a nitrocellulose membrane. The membrane was blocked with PBST containing 5% milk at room temperature for 1 h, and incubated with antibodies (diluted at 1:4000) for 1 h at room temperature with rocking, followed by three times of washes with PBST. Membranes were incubated with HRP-labeled Goat Anti-Rat IgG(H+L) (Beyotime; 1:10000) for 1 h at room temperature with rocking, followed by three times of washes with PBST. Membranes were incubated briefly in Western lighting ECL substrate system (Thermo Scientific, USA) before exposure to KODAK X-OMAT AR X-ray film (Eastman Kodak, USA).

### PAMP Binding Assay

The PAMP binding assay was performed according to previously description with some modification (35). Briefly, 20 μg of LPS, PGN, β-glucan, mannose (Sigma-Aldrich) in 100 μL of carbonate-bicarbonate buffer (50 mmol L^-1^, pH = 9.6) were used to coat 96-well microliter plates (Costar) and incubated at 37°C for 3 h. The wells were washed three times with PBST for 5 min per time and then blocked with 200 μL of 5% BSA in PBS at 37°C for 1 h. The plate was washed three times and 100 μL of r*Cg*DM9CP-4 solution in different concentration was added to the wells with the presence of 5 mmol L^-1^ CaCl_2_ and 0.1 mg mL^-1^ BSA. After incubation at 37°C for 1 h, the plate was washed three times with PBST and then the bound protein was detected immunochemically. Rat anti-His taq monoclonal antibody (1:1,000 dilution in TBS) was added as primary antibody and incubated at 37°C for 1 h, and then incubated with 100 μL of goat-anti-rat Ig-alkaline phosphatase conjugate (1:4,000 dilution in PBS) as the second antibody. The wells were washed four times with TBST for 15 min after each incubation and then incubated with 100 μL of 0.1% (w/v) nitrophenyl phosphate (pNNP, Sigma) in 50 mmol L^-1^ carbonate buffer (pH= 9.8) containing 0.5 mmol L^-1^ MgCl_2_ at room temperature in dark for 15 min. The reaction was stopped by adding 50 μL of 2 mol L^-1^ H_2_SO_4_ per well and the absorbance was measured with an ELISA reader at 450 nm (Molecular Devices). The wells filled with 100 μL of TBS were used as negative control (blank). The apparent dissociation constant (Kd) values were calculated using Prism 5.00 software (GraphPad software) with a one-site binding model and nonlinear regression analysis where A is the absorbance at 450 nm.

### Microbial Binding Assay

Microbial binding activity was measured according to previous report with some modification (36). Gram-positive (*S. aureus* and *M. luteus*), gram-negative bacteria (*V. splendidus*, *V. anguillarum and E. coli*) and fungi (*P. pastoris* and *Y. lipolytica*) were used to exam the microbial binding activity of rCgDM9CP-4. The microbes were suspended in TBS (OD_600_ = 2), and incubated with rCgDM9CP-4 (100 μg mL^-1^) under rotation slightly at 4°C overnight. After three times washing, the bound proteins were dissociated from the microorganisms by loading buffer and analyzed by Western blot as described above. rTrx was employed as negative control, and the purified protein was set as positive control.

### Microbial Agglutination Assay

The overnight cultured Gram-positive bacteria (*S. aureus* and *M. luteus)*, Gram-negative bacteria (*E. coli*) and fungi (*P. pastoris*) were suspended in TBS buffer at 2×10^9^ cells mL^-1^. An aliquot of 50 mL microbial suspension was incubated with 50 μL purified protein (1 mg mL^-1^) at room temperature under rotation for 30 min. rTrx protein was set as control, and 50 μL TBS was used as blank control. After three times of washing with PBS, 50 μL FITC-labeled rat anti-his taq antibody (diluted 1:1000) was used to suspend the microbial suspension, and incubated in dark for 20 min. The agglutination was observed under fluorescence microscopy (Olympus).

### Immunochemistry and Flow Cytometry Analysis of *Cg*DM9CP-4

Immunohistochemistry (IHC) and immunocytochemistry (ICC) assays were conducted as previous description with some modification (24, 37). For IHC, tissues were fixed using Bouin’s fixative (Saturated picric acid solution: formaldehyde: glacial acetic acid=15:5:1) at room temperature for 24 h. Tissue samples were then faded in 70% ethanol for 2 h for 2–4 times and then dehydrated in 80, 95, and 100% successive ethanol baths. After dehydrated with Xylene/ethanol (1:1), Xylene, respectively, all the tissue samples were embedded in paraffin, and the cross-sections were prepared by RM-2016 microtome (LEIKA, Germany). Paraffin was eliminated in Xylene bath and the sections were rehydrated in successive 95 to 30% ethanol baths and finally in distilled water. Antigens were refolded in sodium citrate-hydrochloric acid buffer. For ICC, hemocytes were obtained from healthy oysters after one week culture in filtered aerated seawater at 18°C and immediately centrifuged at 800 × *g*, 4°C for 10 min. Modified-Leibovitz L-15 mediums (M-L15, Gibco) according to the previous description were used to suspend the hemocytes, and the suspension was added into cell culture dishes (38). After incubated at room temperature for 3 h, the supernatant was discarded and 4% PFA (Paraformaldehyde diluted in TBS) was used to fix the hemocytes for 15 min. Then the sections/dishes were blocked with 500 μL of 3% BSA in PBS at 37°C for 30 min, the supernatant was removed and were incubated with 500 μL antibody of *Cg*DM9CP-4 (diluted 1:500 in blocking buffer) as the primary antibody at 37°C for 1 h. After washing three times with PBST, the samples were incubated with Alexa Fluor 488-labeled goat-anti-rat antibody (diluted 1:1000 in PBST) as the second antibody at 37°C for 1 h. After another three times of washing with PBST, DAPI (diluted 1:10,000 in PBS) was added to stain the nucleus and DIL (diluted 1:10,000 in PBS) to stain the membrane. After the last three times of wash, samples were observed under fluorescence microscopy (Olympus) and Laser Scan Confocal Microscope (ZEISS).

For flow cytometry analysis, hemocytes from six oysters were collected and centrifuged at 800 × *g*, 4°C for 10 min immediately. After three times of wash in M-L15, same volume of 4% PFA was added to fix the hemocytes for 5 min. After three times of wash in TBST, the hemocytes were then blocked with 500 μL of 3% BSA in PBS at room temperature for 1 h, and then the supernatant was removed and the hemocytes were incubated with 500 μL antibody of *Cg*DM9CP-4 (diluted 1:1,000 in 3% BSA) as the primary antibody at room temperature for 1 h. The hemocytes were then incubated with Alexa Fluor 488-labeled goat-anti-rat antibody (diluted 1:1,000 in 3% BSA) as the second antibody at 37°C for 1 h after three times washing. The expression pattern of CgDM9CP-4 was analyzed on an FACS Arial II flow cytometer (Becton, Dickinson and Company).

To analyze hemocytes form the gills, gills from three oysters were collected and cut into small pieces, then soaked in M-L15 for 10 min to extract the hemocytes from gill. Flow cytometry analysis and immunocytochemistry were then performed as described above.

### Detection of Phagocytosis, ROS and Cytokine Expressions in *Cg*DM9CP-4-Positive Hemocytes by Flow Cytometry

Phagocytosis assay was performed following the previous description (39). Briefly, hemocytes were incubated with Latex beads (2 μm, Sigma) at room temperature for 1 h with rotation, and wash three times with M-L15. Intracellular ROS level was detected using the peroxide-sensitive fluorescent probe DCFH-DA (Beyotime, China) as substrate according to the previous report with some modification (40). The hemocytes were then incubated with 500 μL *Cg*DM9CP-4 antibody. For intracellular cytokine expression, antibody of *Cg*TNF-α (CGI_10008787 or CGI_10008788), *Cg*IFNLP or *Cg*IL17-5 (diluted 1:1,000 in 3% BSA) were added together with *Cg*DM9CP-4 antibody as the primary antibodies. The hemocytes were then incubated with Alexa Fluor 488-labeled goat-anti-rat antibody and Alexa Fluor 594-labeled goat-anti-mouse antibodies (diluted 1:1000 in 3% BSA) as the second antibody at 37°C for 1 h after three times washing. The phagocytic rate and ROS level, as well as the expression levels of cytokines in *Cg*DM9CP-4-positive hemocytes were analyzed on an FACS Arial II flow cytometer (Becton, Dickinson and Company).

### The mRNA Expression of *Cg*DM9CP-4 in Phagocytes and Non-Phagocytes

Phagocytes and non-phagocytes were separated following the previous description (22). In brief, hemolymph was collected from one hundred oysters and immediately centrifuged at 800 × *g*, 4°C for 10 min to harvest the hemocytes. The hemocytes were suspended in resuspension and incubated with Latex beads (2 μm, Sigma) at room temperature for 1 h with rotation. The viability of hemocytes was measured by propidium iodide positivity under fluorescence microscopy (Olympus). The single-cell suspensions were sorted by flow cytometer at the sorting efficiency of 90%. The separated phagocytes and non-phagocytes were rechecked for the validity of the cell sorting. The total RNA was extracted from the hemocytes using Trizol reagent. cDNA synthesized and SYBR Green fluorescent qRT-PCR as described above.

### *In Vitro* RNA Interference of Hematopoietic Related Genes

T7 promoter linked primers (**Supplementary Table 1**) were used to amplify *Cg*GATA, *Cg*Runt, *Cg*SCL and *Cg*Notch cDNA fragments from *C. gigas* following the previous description (41). EGFP DNA fragment (657 bp) amplified from pEGFP vector (Clontech, USA) was employed as control. The PCR products were used as templates to synthesize dsRNA by *in vitro* transcription according to the method described by previous reports (42). The RNA integrity was examined by electrophoresis and the concentration was quantified by the absorbance at 260 nm and adjusted to a final concentration of 1 mg mL^-1^. Ninety oysters were randomly divided into six groups with fifteen individuals in each group. dsRNAs (100 μg per oyster) of *Cg*GATA, *Cg*Runt, *Cg*SCL, *Cg*Notch and EGFP were injected into the adductor of each oyster, respectively. The oysters received an injection of 100 μL sterile seawater were set as blank control. Hemolymph samples from six oysters of each group were collected at 24 h post-dsRNA injection for total RNA extraction. The efficiency of gene knock-down was checked using qRT-PCR. The expression level of *Cg*DM9CP-4 was detected by qRT-PCR as described above.

### 5-Ethynyl-2’-Deoxyuridine(EdU) Labeling and *Cg*DM9CP-4-Positive Hemocytes in Gill

EdU labeling assay was performed as previously described (43). In brief, six oysters were cultured with sterile seawater with EdU (Life technologies, 2 mg L^-1^) in an aerated tank for 48 h. Cross sections of gill were made as described above. The slides were then fixed with 4% PFA at room temperature for 15 min. After three times washing with 3% BSA in TBS, 0.1% Triton^®^ X-100 in TBS was used to treat the samples at room temperature for 10 min. *Cg*DM9CP-4 positive cells were stained as mentioned above and after the final three times washing with TBS (containing 3% BSA), 0.1% Triton^®^ X-100 in TBS was used to treat the samples at room temperature for 10 min. Samples were then incubated with the 1 × Click-iT™ Reaction Buffer (provided with the kit) for 30 min, and washed thoroughly with PBS (3 × 15 min). EdU were analyzed on an FACS Arial II flow cytometer (Becton, Dickinson and Company, USA). For the new generated hemocytes in gill, slices were incubated with DAPI (diluted 1:1,000 in PBST) for 5 min, washed extensively with PBS, mounted with 80% glycerin, and monitored under a Laser Scan Confocal Microscope (ZEISS).

### Statistical Analysis

All the data were expressed as mean ± standard deviation (N = 3 or 4), and analyzed by Statistical Package for Social Sciences (SPSS) 18.0. The significant differences among groups were tested by one-way analysis of variance (ANOVA) and multiple comparisons. Statistically significant differences were designated at *p* < 0.05 and extremely significant at *p* < 0.01.

## Result

### Sequence Characteristics and Multiple Alignment of *Cg*DM9CP-4

**Figure 1:**
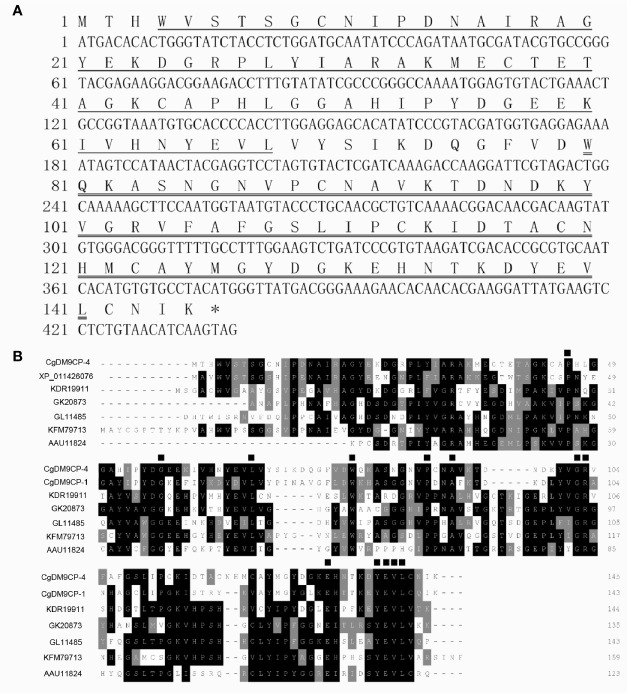
Sequence features of *Cg*DM9CP-4 and multiple sequence alignment of DM9CPs. **(A)** The nucleotide and the deduced amino acid sequence of *Cg*DM9CP-4. The first putative DM9 domain was underlined, and the second was double-underlined. The asterisks indicated the stop codon. **(B)** Multiple sequence alignment by ClustalW of *Cg*DM9CP-4 with other DM9CPs. Sequences filled in black showed the conserved amino acid residues, and similar amino acids are in grey. The conserved amino acids involved in mannose binding were marked with ▪.

The sequence of *Cg*DM9CP-4 was retrieved from NCBI (GenBank Accession No. XP_011421715). A cDNA fragment of 438 bp was amplified by one pair of gene specific primers. The open reading frame (ORF) of *Cg*DM9CP-4 encodes a polypeptide of 145 amino acids with a predicted molecular mass of 16 kDa. There were only two tandem DM9 domains in *Cg*DM9CP-4 identified by SMART analysis (**Figure 1A**).

BLAST analysis revealed that the deduced amino acid sequence of *Cg*DM9CP-4 shared high similarities with other known DM9CPs. *Cg*DM9CP-4 shared 63% similarity with Natterin-4 from *C. gigas* (XP_011426076), 38% with GL11485 from *Drosophila persimilis*, 42% with Natterin-3 from *Zootermopsis nevadensis* (KDR19911), 35% with Natterin-3 from *Thalassophryne nattereri* (AAU11824), 40% with Natterin-4 from *Stegodyphus mimosarum* (KFM79713) and 37% with GK20873 from *Drosophila willistoni* (XP_002061352) (**Figure 1B**). The conserved amino acid sites were identified by multiple alignment, such as K^43^, P^46^, G^58^, L^69^, W^81^, P^90^, A^93^, G^102^, R^103^, E^132^. A conserved signature sequence involved in pattern recognition was characterized from Y^138^ to L^141^ (**Figure 1B**).

### Tissue Distribution and Temporary Expression Patterns of *Cg*DM9CP-4 mRNA After Immune Challenges

**Figure 2:**
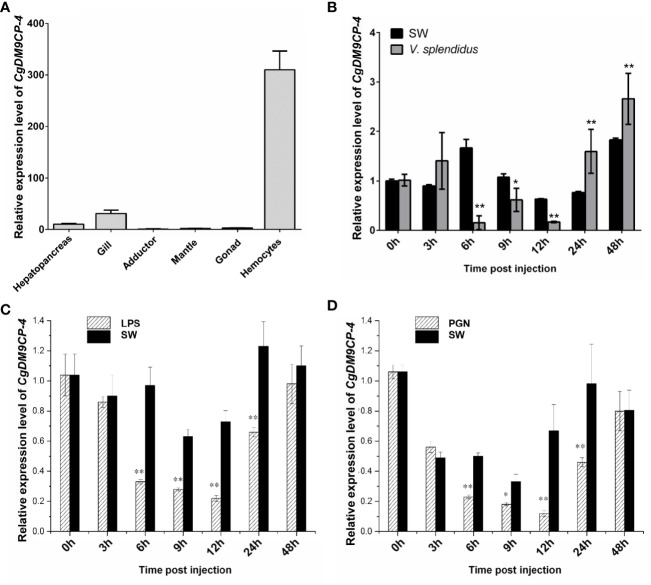
Expression patterns of *Cg*DM9CP-4 mRNA in different tissues and post stimulation by real-time PCR. **(A)** Comparison of the expression level of *Cg*DM9CP-4 mRNA (relative to *Cg*EF) among different tissues was normalized to adductor. Vertical bars represent the mean ± S.D. (N = 4). **(B–D)** Comparison of the level of *Cg*DM9CP-4 mRNA (relative to *Cg*EF) post *V. splendidus* **(B)**, LPS **(C)** and PGN **(D)** were normalized to 0 h. Vertical bars represent the mean ± S.D. (N = 4). **P < 0.01, *P < 0.05.

*Cg*DM9CP-4 mRNA could be detected in all the tested tissues by qRT-PCR, including hemocytes, gonad, muscle, mantle, gill, and hepatopancreas (**Figure 2A**). The highest expression level was detected in hemocytes and gill, which was 310.15-fold (*p* < 0.01) and 30.78-fold (*p* < 0.01) higher than that of adductor, respectively.

The temporal expression level of *Cg*DM9CP-4 mRNA was then examined in oyster hemocytes at 0, 3, 6, 9, 12, 24 and 48 h after *V. splendidus* stimulation (**Figure 2B**). The transcripts of *Cg*DM9CP-4 in circulating hemocytes was significantly decreased at 6 (9.5-fold, *p* < 0.01), 9 (1.8-fold, *p* < 0.05) and 12 h (3.2-fold, *p* < 0.01) compared to blank group, respectively (**Figure 2B**), followed by a slight increase of at 24 (2.1-fold, *p* < 0.01) and 48 h (1.4-fold, *p* < 0.01), respectively (**Figure 2B**).

Finally, the temporal expression of *Cg*DM9CP-4 mRNA in circulating hemocytes post LPS and PGN stimulation was examined (**Figures 2C, D**). The transcript of *Cg*DM9CP-4 was significantly decreased at 6 (2.72-fold, *p* < 0.01), 9 (2.24-fold, *p* < 0.01), 12 (2.65-fold, *p* < 0.01) and 24 h (1.7-fold, *p* < 0.01), and raised up to the original level at 48 h compared with 0 h after LPS stimulation (**Figure 2C**). In the PGN stimulation group, *Cg*DM9CP-4 transcript level was decreased at 6 (2.12-fold, *p* < 0.01), 9 (1.66-fold, *p* < 0.01), 12 (4.45-fold, *p* < 0.01) and 24 h (1.83-fold, *p* < 0.01), and recovered to normal level at 48 h (**Figure 2D**).

### The Recombinant Protein of *Cg*DM9CP-4 and Production of Polyclonal Antibody

**Figure 3:**
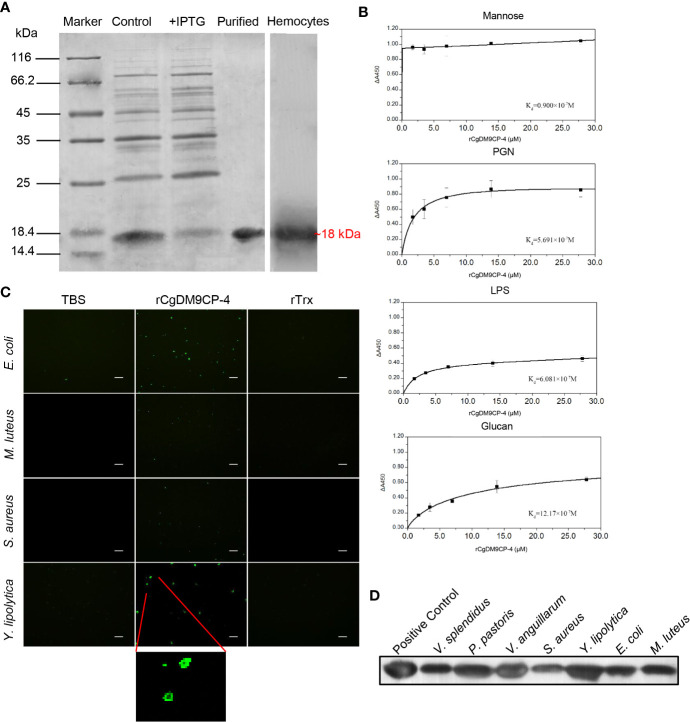
Recombinant protein of *Cg*DM9CP-4 preparation and verification of the pattern recognition function. **(A)** SDS-PAGE analysis of purified r*Cg*DM9CP-4 protein and western-blot using the polyclonal antibody against *Cg*DM9CP-4 in oyster hemocytes sample. **(B–D)** PAMPs binding **(B)**, bacteria agglutination **(C)** and bacteria binding **(D)** activity of r*Cg*DM9CP-4. ELISA assay was performed to determine the binding dissociated constant of r*Cg*DM9CP-4 (0–12 μM) with LPS, Mannose, PGN and Glucan. Data are shown as the mean ± S.D. (N = 3). The data were curve-fitted using a single-site binding model. Bacteria agglutination activity of r*Cg*DM9CP-4 was visualized by immunostaining by using FITC-labeled rat anti-his taq antibody after incubation of r*Cg*DM9CP-4 with various microorganisms. Binding of r*Cg*DM9CP-4 to Gram-positive, Gram-negative bacteria and fungi was measured by Western blotting analysis.

After Coomassie blue staining, a distinct band of r*Cg*DM9CP-4 was revealed with a molecular weight of ~18 kDa, which was consistent with the predicted molecular mass (**Figure 3A**). The purified r*Cg*DM9CP-34 was employed to prepare polyclonal antibody and the following functional verification assay.

A clear reaction band was revealed in hemocytes by western blot to identify the specificity of the polyclonal antibody (**Figure 3A**). Pre-immune serum from mice was set as negative control group with no visible reaction band (data not shown).

### PAMPs Binding Activity, Microbe Binding Spectrum and Microbial Agglutinating Activity of r*Cg*DM9CP-4

The PAMPs binding activity of r*Cg*DM9CP-4 was evaluated by ELISA assay. r*Cg*DM9CP-4 could bind LPS, PGN and β-glucan in a dose-dependent manner. r*Cg*DM9CP-4 exhibited highly binding activity towards Mannose from low concentration (**Figure 3B**). In addition, r*Cg*DM9CP-4 showed higher affinities to Mannose and PGN (K_d_=0.9×10^-7^M and 5.691×10^-7^M) while relative lower affinities to LPS and β-glucan (K_d_=6.081×10^-7^M and 12.17×10^-7^M). As a control, no binding affinity was found in the group of rTrx (data not shown).

Gram-positive (*S. aureus* and *M. luteus*) and gram-negative (*E. coli*) bacteria, as well as fungi (*Y. lipolytica*) were used to test the agglutination activity of r*Cg*DM9CP-4. r*Cg*DM9CP-4 exhibited no agglutinating activities towards any of the microorganisms. Instead, r*Cg*DM9CP-4 showed binding activity to all the tested microorganisms as clear signals of r*Cg*DM9CP-4 were detected on the membranes (**Figure 3C**).

Western blotting was then carried out to analyze the binding capability of r*Cg*DM9CP-4 to gram-positive bacteria, gram-negative bacteria, and fungi. Purified r*Cg*DM9CP-4 was set as positive control which showed a clear band (**Figure 3D**). No significant signal was detected in the negative control (data not shown). r*Cg*DM9CP-4 was specifically detected in all seven microorganisms [gram-positive (*S. aureus* and *M. luteus*), gram-negative (*V. splendidus*, *V. anguillarum and E. coli*) and fungi (*P. pastoris* and *Y. lipolytica*)] groups (**Figure 3D**). The results indicated that r*Cg*DM9CP-4 displayed stronger binding affinity to *P. pastoris*, *Y. lipolytica and V. anguillarum*, lower binding affinity to *S. aureus*, *M. luteus*, *E. coli*, and *V. splendidus*, respectively.

### Localization of *Cg*DM9CP-4 in Tissues and Hemocytes

**Figure 4:**
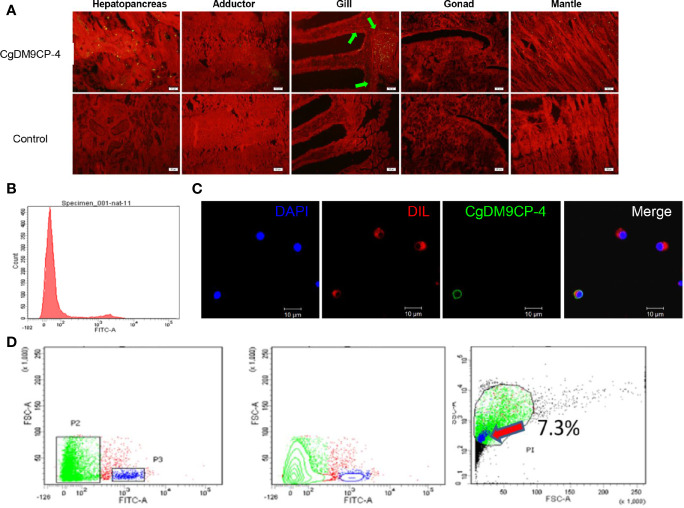
Immunohistochemistry, immunofluorescence and flow cytometry analysis of the localization of *Cg*DM9CP-4 protein. **(A)** Distribution of *Cg*DM9CP-4 was visualized by Alexa Fluor 488-labeled goat-anti-rat antibody (upper panel), the rat pre-immuned serum was used as control (lower panel), the tissues were stained with Evans blue dye (red). Bar= 50 μm. **(B)** Expression of *Cg*DM9CP-4 on the membrane of hemocytes as detected by flow cytometry. **(C)** Localization of *Cg*DM9CP-4 in hemocytes. Nucleus was stained with DAPI (blue), *Cg*DM9CP-4 (green) and the cell membrane were stained with CM-DIL (red). Bar= 10 μm. **(D)** Distinct gating by forward scatter (FSC) and Alexa Fluor 488-labeling and (left), contour by FSC and Alexa Fluor 488-labeling showing the specific group of *Cg*DM9CP-4-positive hemocytes (middle), size and granularity of *Cg*DM9CP-4-positive hemocytes were analyzed by Side scatter (SSC) and FSC (right).

Immunohistochemistry assay was used to detect the localization of *Cg*DM9CP-4 protein in different tissues and hemocytes. *Cg*DM9CP-4 protein expression could be detected in mantle, gill, and hepatopancreas (**Figure 4A**). Specifically, *Cg*DM9CP-4 was highly expressed at the base of the gill where filaments were projected out (**Figure 4A**). In hemocytes, *Cg*DM9CP-4 was detected to localize on the membrane of hemocytes with a cell specificity distribution (**Figures 4B, C**). Hemocytes from oyster *C. gigas* could be clearly separated into two groups by the polyclonal antibody of *Cg*DM9CP-4 (**Figure 4B**). *Cg*DM9CP-4-positive hemocytes were small in size and had a higher nuclear-cytoplasmic ratio (**Figure 4C**). Specifically, *Cg*DM9CP-4-positive hemocytes accounted for 7.3% of total hemocytes, which were smaller in size and less in granularity compared with *Cg*DM9CP-4- negative cells (**Figure 4D**).

### Immune Capacity of *Cg*DM9CP-4-Positive Hemocytes

**Figure 5:**
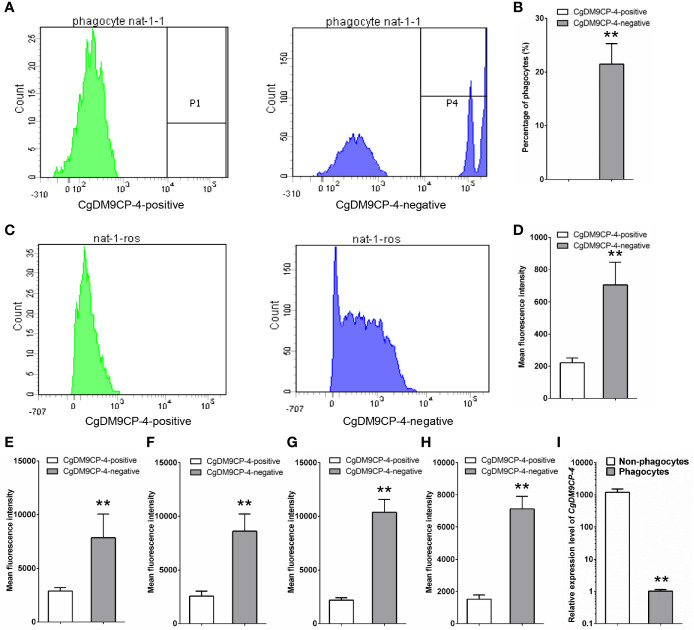
Phagocytic rate, ROS level and cytokine level of *Cg*DM9CP-4-positive hemocytes detected by flow cytometry. **(A)** Phagocytic rate of *Cg*DM9CP-4-positive and -negative hemocytes. **(B)** Quantification based on **(A). (C)** ROS level of *Cg*DM9CP-4-positive and -negative hemocytes. **(D)** Quantification based on **(C) (E–H)** Relative protein levels of TNF (E:CGI_10008787, F: CGI_10008788), *Cg*IFNLP **(G)** and *Cg*IL17-5 **(H)** in *Cg*DM9CP-4-positive and -negative hemocytes. **(I)** Expression level of *Cg*DM9CP-4 mRNA in sorted phagocytes and non-phagocytes. Data represent the mean ± S.D. (N = 3). **P < 0.01.

The immune capacity of *Cg*DM9CP-4-positive and -negative hemocytes was evaluated by flow cytometry. The phagocytic rate of *Cg*DM9CP-4-positive hemocytes was 0 and the phagocytic rate of the rest negative hemocytes was 21.5% (**Figures 5A, B**). The relative ROS level of *Cg*DM9CP-4-positive hemocytes indicated by mean fluorescence intensity was 203, which was much lower than that in *Cg*DM9CP-4-negative hemocytes (706, *p* < 0.01) (**Figures 5C, D**).

Then flow cytometry methods was used to determine the protein levels of different cytokines, including *Cg*TNF-α (CGI_10008787, CGI_10008788), *Cg*IFNLP and *Cg*IL17-5, in *Cg*DM9CP-4-positive and -negative hemocytes. The relative protein levels of CGI_10008787, CGI_10008788, IFNLP, IL-17 indicated by mean fluorescence intensity were 2566 ± 472, 2195 ± 231, 2895 ± 316 and 1523 ± 265 in *Cg*DM9CP-4-positive hemocytes respectively (**Figures 5E–H**), which were significantly lower than that in *Cg*DM9CP-4-negative hemocytes (CGI_10008787: 8602 ± 1625, *p* < 0.01; CGI_10008788: 10,370 ± 1232, *p* < 0.01; IFNLP: 7839 ± 2207, *p* < 0.01; IL-17: 7187 ± 897, *p* < 0.01) (**Figures 5E–H**).

Finally, the phagocytes and non-phagocytes from *C. gigas* hemocytes were separated by fluorescence activated cell sorting using flow cytometry (**Figure S1**). The expression level of *Cg*DM9CP-4 in non-phagocytes (which accounted for ~82% of total hemocytes) was 1190.3-fold (*p* < 0.01) higher than that in phagocytes (**Figure 5I**). All these results demonstrated that *Cg*DM9CP-4-positive hemocytes exhibited lower immune capacity.

### Expression Levels of *Cg*DM9CP-4 After Knockdown of Hematopoietic Transcription Factors and Co-Localization of *Cg*DM9CP-4 With New-Born Hemocytes

As the gill of *C. gigas* has been reported to be the hematopoietic tissue, we then examined the protein expression of *Cg*DM9CP-4 in hemocytes from the gill. *Cg*DM9CP-4-positive hemocytes was more abundant in gill than in hemocytes (**Figures S2A–C**) and a subpopulation of hemocytes with much stronger *Cg*DM9CP-4 level was also identified from gill (**Figure S2A**). In addition, after *V. splendidus* stimulation, the percentage of *Cg*DM9CP-4-positive hemocytes was firstly decreased at 3 and 6 h, then increased at 9, 12, and 24 h, and finally fell down to normal level at 48 h (**Figure S3A**). The expression level of *Cg*DM9CP-4 in gill also showed a trend of increase and then decrease, finally back to normal trend after *V. splendidus* stimulation (**Figure S3B**).

**Figure 6:**
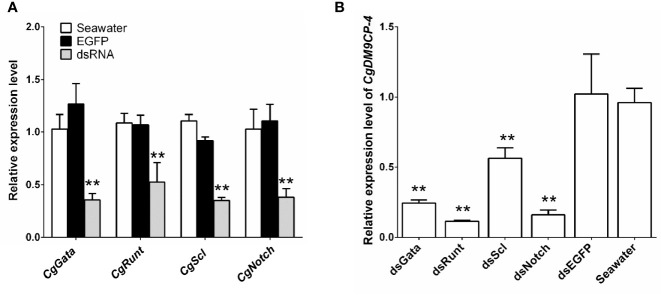
Expression level of *Cg*DM9CP-4 mRNA in hemocytes after knockdown of hematopoietic factors. **(A)** RNAi knockdown efficiency of *Cg*GATA, *Cg*Runt, *Cg*SCL, and *Cg*Notch. **(B)** *Cg*DM9CP-4 mRNA levels in hemocytes after knockdown *Cg*GATA, *Cg*Runt, *Cg*SCL, and *Cg*Notch. Data represent the mean ± S.D. (N = 3). **P < 0.01.

Then we knockdown the mRNA expression levels of hematopoietic factors *Cg*GATA, *Cg*Runt, *Cg*SCL and *Cg*Notch by using RNAi. After 24 h of dsRNA injection, the expression levels of *Cg*GATA, *Cg*Runt, *Cg*SCL and *Cg*Notch in hemocytes (**Figure 6A**), as well as the expression levels of *Cg*DM9CP-4 in hemocytes from *Cg*GATA (0.24-fold, *p* < 0.01), *Cg*Runt (0.11-fold, *p* < 0.01), *Cg*SCL (0.56-fold, *p* < 0.01) and *Cg*Notch (0.16-fold, *p* < 0.01) knockdown groups were all significantly down-regulated in comparison with that in dsEGFP group, respectively (**Figure 6B**).

**Figure 7:**
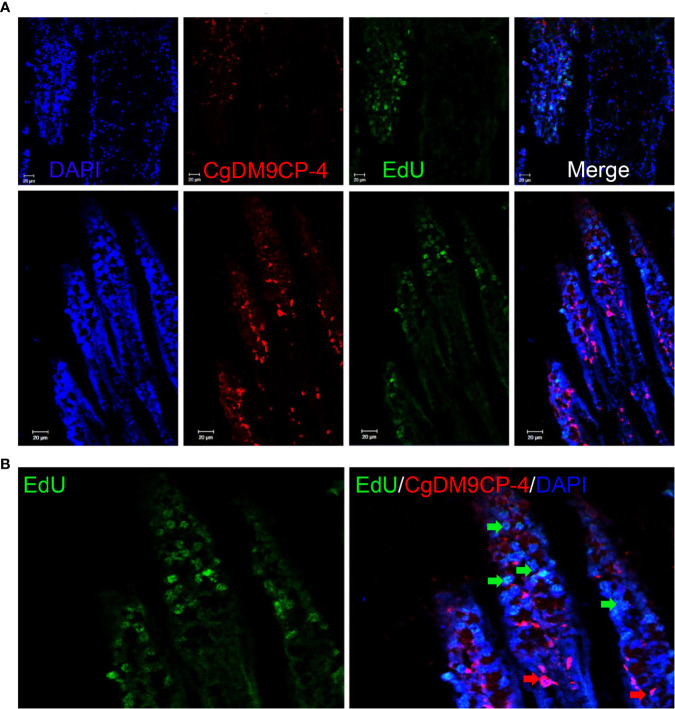
Localization of *Cg*DM9CP-4-positive hemocytes and EdU-positive cells in gill. Immunofluorescence of *Cg*DM9CP-4 and EdU in gill sections **(A)** and representative enlarged image **(B)**.

We further performed EdU labeling assay in oyster gill to investigate the potential relationship between *Cg*DM9CP-4-positive hemocytes and new-born hemocytes. After 48 h of EdU incubation, *Cg*DM9CP-4-positive hemocytes were found to appear at the lumen of gills where hematopoietic stem cells were located on top of the filaments (**Figures 7A, B**).

## Discussion

DM9CPs refer to a new family of PRR with tandem DM9 domains that identified from the Pacific oyster *C. gigas.* Although DM9CPs have been shown to mediate the innate immune recognition through binding to various PAMPs, their function in cellular immunity and hemocyte-specific expression has not been investigated. Here, we identified a novel hemocyte-specific role of *Cg*DM9DCP-4 from *C. gigas.* Specifically, it is found that *Cg*DM9DCP-4 is not limited to the common function as a PRR with other DM9CPs, it is also localized on the membrane of oyster pro-homocytes. The *Cg*DM9DCP-4-positive hemocytes were small in size and exhibited less immune capacity. Furthermore, *Cg*DM9DCP-4-positive hemocytes were more abundant at the lumen of gills that downstream of the hematopoietic stem cells. The present study suggests that *Cg*DM9DCP-4 is a hemocyte-specific PRR that possibly involved in the immune recognition of pro-hemocyte. Future studies to dissect the role *Cg*DM9DCP-4 in pro-hemocyte maturation would be helpful to develop new insights into the hematopoiesis and hemocyte classification in oyster.

DM9 domain is found to tandem double or quadruple repeated and is barely in combination with other domains (29). The function of DM9 domain is previously unclear and the researchers predicted that DM9CP is involved in regulatory interactions during local immune response and DM9 domain is similar to lectin domain that facilitate cell membrane binding (44). In the present study, the ORF of *Cg*DM9CP-4 cDNA encoded a polypeptide of 145 amino acids with two tandem DM9 domains. The amino acid sequences of *Cg*DM9CP-4 shared high similarity with other reported DM9CPs with two DM9 domains. The signature sequences identified from *Cg*DM9CP-1 that involved in the pattern recognition is also conserved in *Cg*DM9CP-4 (31), which indicated *Cg*DM9CP-4 might also share the common feature of this novel PRR family in *C. gigas*. Pathogen recognition is a critical link in invertebrate innate immune system for detecting the invading non-self. DM9CPs have been reported to participate in the immune response against pathogen infection (45–47). In the present study, r*Cg*DM9CP-4 could bind to various PAMPs, including LPS, PGN, β-glucan and mannose, suggesting that *Cg*DM9CP-4 was involved in the innate immune recognition and served as a PRR. Consistent with *Cg*DM9CP-1, *Cg*DM9CP-4 exhibited higher binding affinity towards mannose, which favored that this DM9CP may be a family of mannose specific PRR. Two mannose-binding sites are identified from *Cg*DM9CP-1 and multiple amino acid residues, especially D^22^ and K^43^, are involved in the mannose binding activity (31). Interestingly, K^43^ was found to be highly conserved in both *Cg*DM9CP-2 and *Cg*DM9CP-4 (33). However, D^22^ is replaced by G^22^ and E^22^ in *Cg*DM9CP-2 and *Cg*DM9CP-4, respectively (33). In another uncharacterized DM9CP from *C. gigas*, they were E^22^ and K^43^, respectively. All these results demonstrated that DM9CPs from *C. gigas* shared the conserved K^43^ for their mannose binding activity and the other amino acid residues maybe involved in their specific functions. Indeed, r*Cg*DM9CP-4 could also bind to various microorganisms and higher affinity towards fungi. Collectively, all these results indicated that *Cg*DM9CP-4 shared the common features of DM9CP family in mannose binding and showed a broad binding spectrum towards PAMPs and microorganisms.

Accumulating evidences have demonstrated that various invertebrate PRRs are involved in agglutinating and antibacterial activities, serve as opsonins to promote phagocytosis of hemocytes (48–50). To our surprise, unlike most of the identified PRRs such as C-type Lectins *Cg*CLec-3 and *Cg*CLec-4, and *Cg*DM9CP-1, *Cg*DM9CP-2 from *C. gigas* (31, 33, 51, 52), r*Cg*DM9CP-4 did not exhibit any bacteria agglutination and killing activities. Considering the lower expression level of *Cg*DM9CP-4 in hemocytes compared to *Cg*DM9DCP-1 (34), the function and consequence after *Cg*DM9CP-4 binding of the invading pathogens are still unclear.

Hemocytes are considered to be the key component in the immune system in lower invertebrates (53). In the present study, the *Cg*DM9CP-4 transcript levels were decreased in circulating hemocytes in response to *V. splendidus* and PAMPs challenge. The results were different from the expression patterns of most other PRRs, such as C-type lectins, Toll-like receptors, which are usually increased post pathogen invasion (10, 34, 54, 55). It has been reported that the transcripts of *Cg*Defhs decrease after immune challenge in circulating hemocytes but increase at the injection site (56). Considering the pathogen recognition ability of *Cg*DM9CP-4, there were possibilities that the down-regulated *Cg*DM9CP-4 expression was due to the migration of hemocytes to the injury site. However, *Cg*DM9CP-4 protein was only detected on the membrane of 7.3% hemocytes. These hemocytes were small in size, with less granularity and higher nuclei-cytoplasmic ratio, which physiologically resembles the features of pro-hemocytes and agranulocytes, and may have the potential to differentiate into immunological hemocytes. In addition, *Cg*DM9CP-4 was high enriched in non-phagocytes (22, 57). These results excluded *Cg*DM9CP-4-positive hemocytes from agranulocytes since agranulocytes from *C. gigas* are reported to be around 27.63% of total hemocytes (58). ROS is important for the differentiation of alternatively activated macrophages and macrophages are the major producers of cytokines such as TNF-α (59, 60). We also found that the ROS level and production of cytokines in *Cg*DM9CP-4-positive hemocytes were much lower than negative hemocytes, suggesting that they were not immunologic effector cells for eliminating the invading pathogens. Taken all together, we speculated that *Cg*DM9CP-4-positive hemocytes might be pro-hemocytes.

The classification of mollusk hemocytes has been impeded at morphological level till now (22). Researchers predicted that the hemocytes of mollusk might have only one single type but at different development stages, which are able to form granules (most likely rich in antimicrobial substances) and degranulate in response to environmental challenge (61, 62). In crayfish, pro-hemocytes were formed in the specific hematopoietic tissue and were able to be released into the circulation (63). In the present study, we found that the mRNA and protein levels of *Cg*DM9CP-4 mRNA highly expressed in hemocytes and gill. In addition, we also found that *Cg*DM9CP-4-positive hemocytes were more abundant in gill than in hemocytes. It has been reported that the hematopoiesis of oyster occurs in gill where progenitor cells differentiate into hemocytes (24). After knockdown of hematopoietic factors, *Cg*GATA, *Cg*Runt, *Cg*SCL, and *Cg*Notch by RNAi, the expression level of *Cg*DM9CP-4 was significantly disrupted. Previous study has proved that RNAi of these genes would decrease the production of hemocytes *via* disturbing the hematopoiesis in gill (41). These data indicated a strong positive correlation of *Cg*DM9CP-4-positive hemocytes with hematopoiesis. Moreover, immunohistochemistry of *Cg*DM9CP-4 in gill and EdU labeling assay further supported our hypothesis that *Cg*DM9CP-4-positive hemocytes appeared in the downstream lumen of gills where hematopoietic stem cells were located on top. *Cg*DM9CP-4-positive hemocytes appeared after the hematopoietic stem cells which is similar to the type 2 cells during crayfish hematopoietic processes (63). Type 2 cells have large nuclei and larger cytoplasm-containing granules and may be the precursors of SGCs and GCs (63). However, the function of *Cg*DM9CP-4 in mediating pro-hemocytes maturation still needs to be further investigated.

## Conclusion

**Figure 8:**
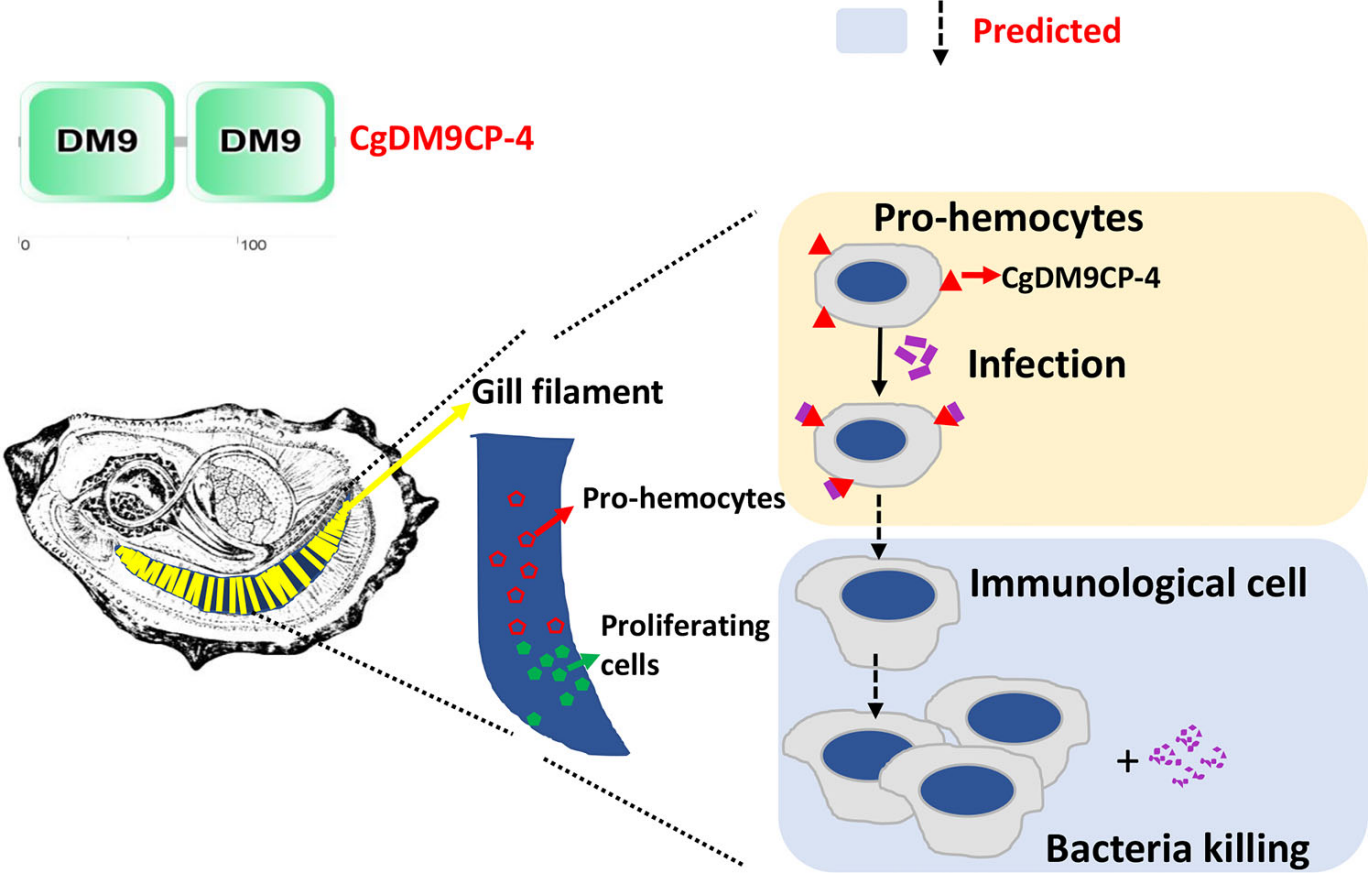
Schematic representation of the mechanism underlying *Cg*DM9CP-4-positve hemocytes in response to stimulation. The green cells in the gill indicate the hematopoietic stem cells that divide and differentiate into pro-hemocytes in gill (Red). *Cg*DM9CP-4 is then recruited on the membrane of pro-hemocytes. When there are invading pathogens, *Cg*DM9CP-4 recognizes the pathogens. The *Cg*DM9CP-4-positve pro-hemocytes may then mature into immunological cells to eliminate the pathogens. At the same time, more pro-hemocytes will be produced and replenished into the circulating hemocytes.

In summary, the result of the study suggests the following model (**Figure 8**): Stem cells firstly divide and differentiate into pro-hemocytes in gill and *Cg*DM9CP-4 is then recruited on the membrane of pro-hemocytes. When there are invading pathogens, *Cg*DM9CP-4 recognizes the pathogens and the *Cg*DM9CP-4-positive pro-hemocytes may subsequently become immunological hemocytes to eliminate the pathogens. At the same time, more pro-hemocytes will be produced and replenished into the circulating hemocytes. *Cg*DM9CP-4 was a novel member of PRR that expressed in undifferentiated pro-hemocytes that recognize the invading pathogens and maybe involved in pro-homocyte maturation. These findings not only merely contribute to the understanding of the DM9 domain and DM9CPs, but also provided new insight into the complex process of hemocyte production and differentiation in mollusk.

## Data Availability Statement

The original contributions presented in the study are included in the article/**Supplementary Material**, further inquiries can be directed to the corresponding author.

## Ethics Statement

The animal study was reviewed and approved by Ethics Committee of the Institute of Oceanology, Chinese Academy of Sciences.

## Author Contributions

ZJ, LW, and LS conceived and designed the experiments and wrote the manuscript. ZJ, SJ, MW, XW, YuL, ZL, XS, and YiL performed the experiments and analyzed the data. All authors revised the manuscript. All authors contributed to the article and approved the submitted version.

## Funding

This research was supported by National Key R&D Program (2018YFD0900502), the National Natural Science Foundation of China (Nos. U1706204, 41961124009), the Outstanding Talents and Innovative teams of Agricultural Scientific Research and Earmarked Fund (CARS-49) from Modern Agro-industry Technology Research System, the Fund for Outstanding Talents and Innovative Team of Agricultural Scientific Research in Ministry of Agriculture, the Research Foundation for Distinguished Professor in Liaoning (XLYC1902012 to LW) and the Climbing Scholar in Liaoning (to LS).

## Conflict of Interest

The authors declare that the research was conducted in the absence of any commercial or financial relationships that could be construed as a potential conflict of interest.
