# Supplementary material for: Identification of a Novel Pattern Recognition Receptor DM9 Domain Containing Protein 4 as a Marker for Pro-Hemocyte of Pacific Oyster Crassostrea gigas
Source: Front Immunol. 2021 Feb 12;11:603270. doi: 10.3389/fimmu.2020.603270 (PMC7907646; doi:10.3389/fimmu.2020.603270)
Supplement: Supplementary file 1 [file DataSheet_1.docx]

Supplementary Material

## Supplementary Figure

##
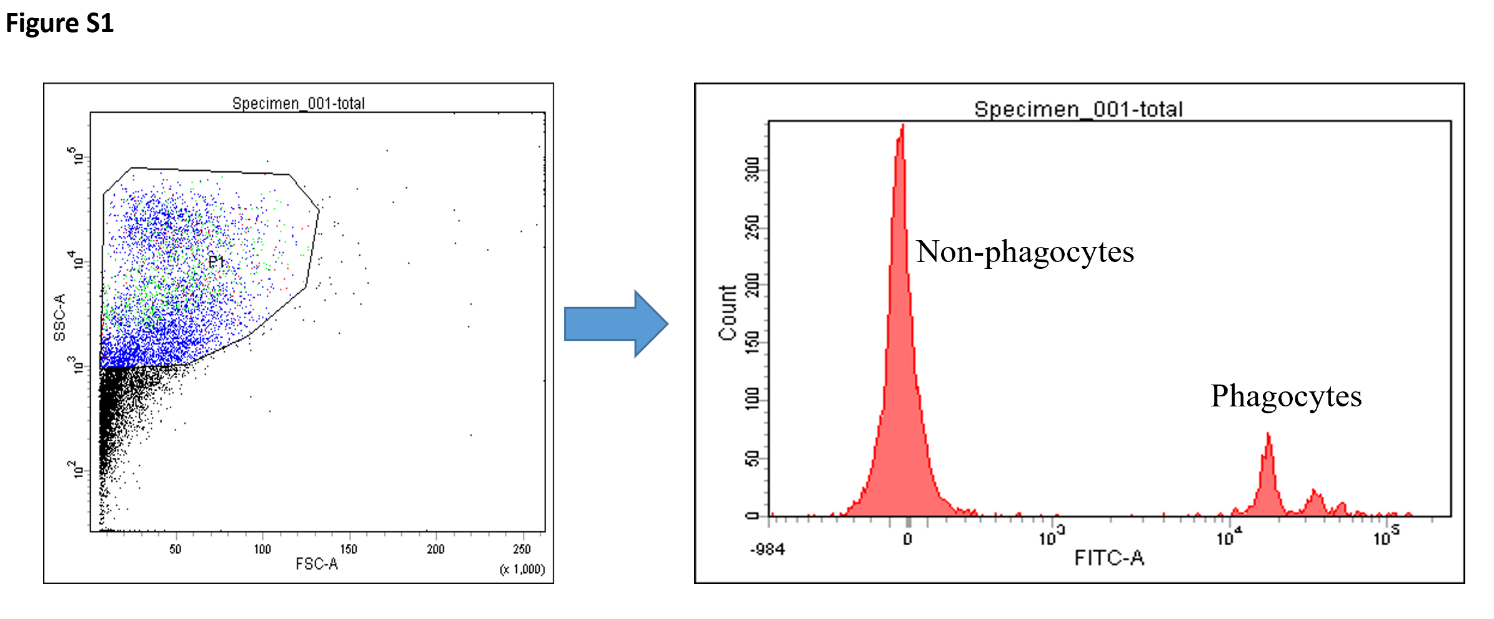


## Figure S1. Fluorescence-activated cell sorting (FACS) of phagocytes and non- phagocytes based on their phagocytosis towards FITC-labeled latex beads.


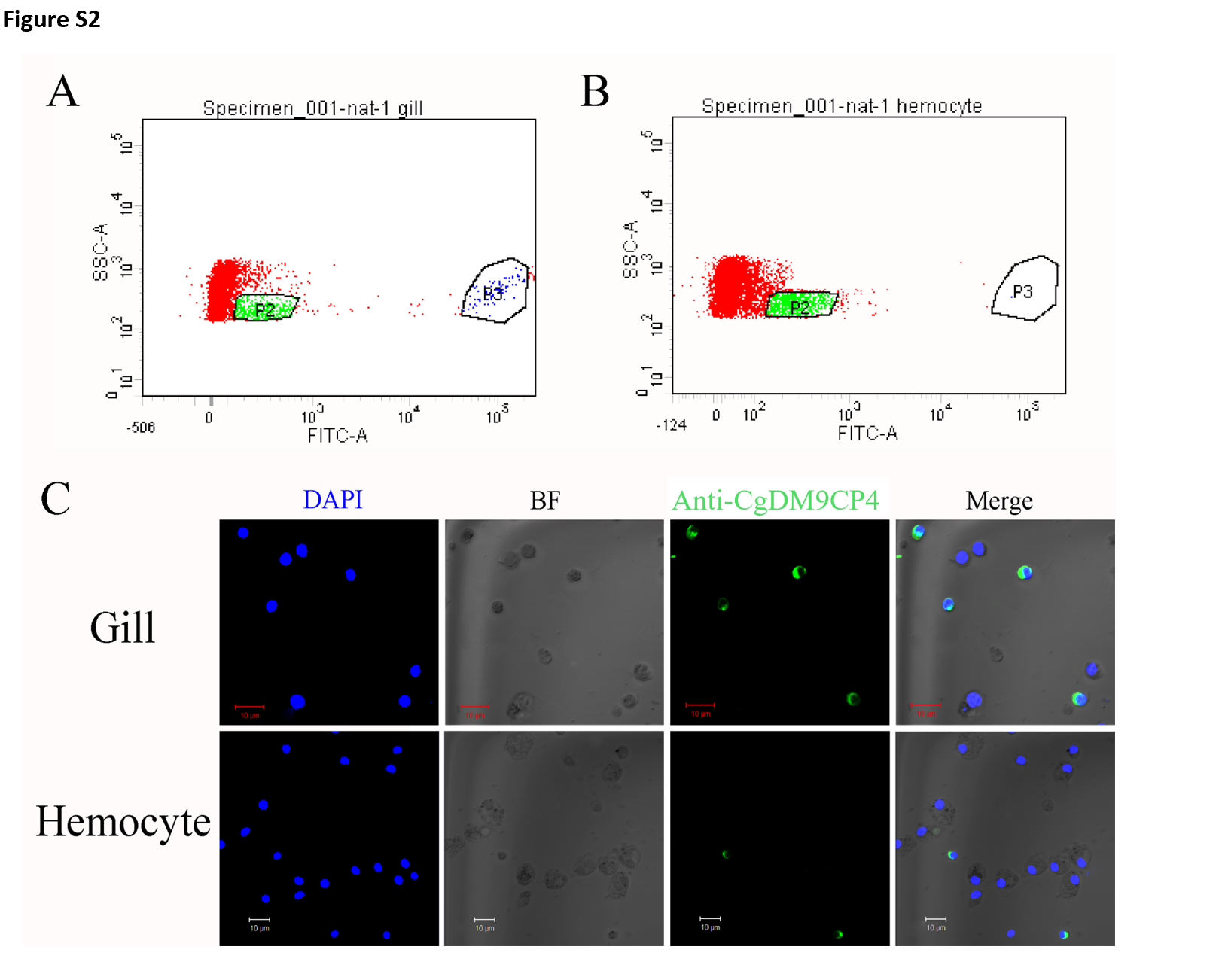


**Figure S2.** Comparison of *Cg*DM9CP-4-positive hemocytes in gill and hemocytes. Analysis of *Cg*DM9CP-4-positive hemocytes from gill (A) and hemocytes (B). (C) Immunofluorescence of *Cg*DM9CP-4 in the hemocytes from gill and isolated from the heart.


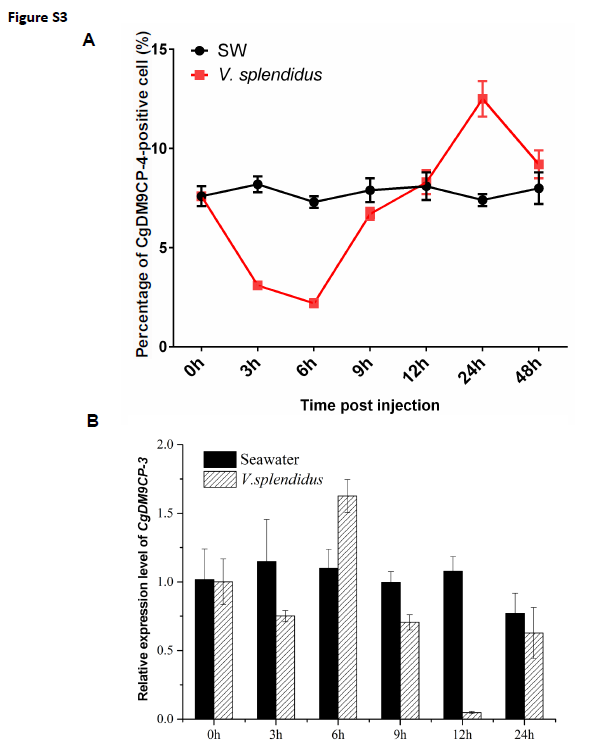


**Figure S3.** Percentage of *Cg*DM9CP-4-positive hemocytes (A) and *Cg*DM9CP-4 mRNA level in gill (B) after *V. splendidus* stimulation.

## Supplementary Table 1

**Primers used in this paper.**

| **Primer** | **Sequence (5’-3’)** |
| --- | --- |

| Oligo (dT)-adaptor  **Clone primers**  P1 (forward)  P2 (reverse)  **RT primers**  P3  P4  **EF primers**  P5 (EF-RTF)  P6 (EF-RTR)  **Recombination primers**  P7 (forward)  P8 (reverse)  **Sequencing primers**  M13 (forward)  RV (reverse) | GGCCACGCGTCGACTAGTACT_17_  ATGACACACTGGGTATCTACCTCTG CCACCTTCCTCCRGTTTRTCA  GTCAAAACGGACAACGACAAGT TCCTTCGTGTTGTGTTCTTTCC  GAGCGTGAACGTGGTATCAC  ACAGCACAGTCAGCCTGTGA  GGAATTCCATATGACACACTGGGTATCTAC CCGCTCGAGCTTGATGTTACAGAGGACTTC  AATTAACCCTCACTAAAGGG  TGCGTCGGCTTTGCTCTG |
| --- | --- |
